# Genome Sequences of Gordonia rubripertincta Bacteriophages AnarQue and Figliar

**DOI:** 10.1128/mra.01085-21

**Published:** 2022-01-20

**Authors:** Ethan Curran, Sabrina E. Callaway, Ramel R. Dumanlang, Anna V. Harshaw, Paula N. Palacio, Yuta Nakamura, Kendra W. Kimberley, James R. Theoret, Earl J. Yoon, Erin J. Windsor, Chelsey C. McKenna

**Affiliations:** a Department of Biological Sciences, College of Southern Nevada, Las Vegas, Nevada, USA; DOE Joint Genome Institute

## Abstract

AnarQue and Figliar are bacteriophages identified from the host bacterium Gordonia rubripertincta NRRL B-16540. AnarQue is circularly permuted and has a length of 61,822 bp; it is assigned to cluster DR. Figliar has a 3′ sticky overhang and a length of 61,147 bp; it is assigned to cluster DJ.

## ANNOUNCEMENT

Two bacteriophages were isolated and characterized as part of the program Science Education Alliance—Phage Hunters Advancing Genomics and Evolutionary Science (SEA-PHAGES) (1). The host bacterium, Gordonia rubripertincta, is a Gram-positive, opportunistic pathogen with potential use for bioremediation (2). The isolation of bacteriophages from this host is useful for investigating bacteriophage diversity. AnarQue was collected from Wetlands Park in Las Vegas (GPS coordinates 36.104529 N, 1115.020891 W). AnarQue was discovered by direct isolation, and it produces clear, circular 3-mm plaques. Figliar was recovered from an enriched soil sample collected from a dog park in Las Vegas (GPS coordinates 36.155333 N, 115.265056 W). Figliar produces small, cloudy 1-mm plaques; however, genome analysis revealed no lysogenic-related genes.

The protocols used for isolation and purification of the bacteriophages and DNA extraction are available from the SEA-PHAGES *Phage Discovery Manual* (3). Soil samples were incubated with phage buffer for 4 h and allowed to settle, and supernatants were sterilized using 0.22-μm filters. For direct isolation, the supernatant was used immediately for the plaque assay. For enriched isolation, 500 μL host bacteria was incubated with the supernatant at 30°C for 72 h prior to the plaque assay. The sample was 0.22-μm filter sterilized and used for the plaque assays. DNA was isolated using the Norgen phage DNA isolation kit modified with five rounds of freeze/thaw (a 4-min freeze in a dry ice-ethanol bath and a 1-min thaw). AnarQue and Figliar were sequenced at the Pittsburgh Bacteriophage Institute using an Illumina MiSeq instrument. Sequencing libraries were generated from the extracted genomic DNA using the New England Biolabs (NEB) Ultra II library preparation kit v3 with 150-base single-end reads, per the manufacturer’s instructions. There was 2,830× coverage for AnarQue, with 1,232,424 reads. There was 793× coverage for Figliar, with 338,736 reads. The sequencing reads were used as inputs for Newbler v2.9 with default settings (4). The contigs produced using Newbler were analyzed using the default settings of Consed v29 (http://www.phrap.org/consed/consed.html) to produce a single contig. Quality control included evaluating for completeness by checking the genome circularization, accuracy by checking for gaps, and determining the genomic termini by searching for overrepresented portions of the DNA (4). The AnarQue genome is 61,822 bp long and has a 68.8% GC content. The Figliar genome is 61,147 bp long and has a 51.5% GC content.

Annotation of AnarQue and Figliar was completed using the following programs: DNA Master v5.23.2 (http://cobamide2.bio.pitt.edu/computer.htm), Starterator v1.2 (https://github.com/SEA-PHAGES/starterator), Phamerator (https://phamerator.org/) (5), PhagesDB BLAST (https://phagesdb.org/blast/) (6), NCBI BLAST (7), PECAAN, GeneMark v2.5p (8), Glimmer v3.02 (9), Aragorn v1.1 and v1.2.38 (10), HHPRED v3.2.0 (11), tRNAscan-SE v2.0 (12), TMHMM v2.0 (13), and SOSUI v1.11 (14). All tools were run with default parameters unless otherwise specified.

The closest related genome to Figliar was bacteriophage Jodelie19 in the same cluster (DJ) with a 97.97% identity match. AnarQue’s closest relative was fellow DR cluster member CloverMinnie, with a 98.22% identity match. Putative functions were assigned to 35 of 90 genes in Figliar and 32 of 86 genes in AnarQue. Neither bacteriophage encoded tRNA or transfer-messenger RNA (tmRNA). The annotation revealed that AnarQue contains a putative endonuclease VII not present in other cluster DR bacteriophages.

### Data availability.

The GenBank and SRA accession numbers for AnarQue are OK216879 and SRR15908344, respectively. The GenBank and SRA accession numbers for Figliar are MZ209301 and SRR15908339, respectively.
